# The influence of somatotype on anaerobic performance

**DOI:** 10.1371/journal.pone.0197761

**Published:** 2018-05-22

**Authors:** Helen Ryan-Stewart, James Faulkner, Simon Jobson

**Affiliations:** Department of Sport, Exercise, and Health, University of Winchester, Winchester, United Kingdom; Nanyang Technological University, SINGAPORE

## Abstract

The link between athlete physique and performance in sports is well established. However, a direct link between somatotype three-numeral rating and anaerobic performance has not yet been reported. The purpose of this study was to assess the relations between somatotype and anaerobic performance using both singular and multivariate analyses. Thirty-six physically active males (mean ± standard deviation age 26.0 ± 9.8 years; body mass 79.5 ± 12.9 kg; height 1.82 ± 0.07 m) were somatotype-rated using the Heath-Carter method. Subjects were assessed for 3 repetition maximum (3 RM) bench press and back squat, and completed a 30-second maximal sprint cycle test. Positive correlations were observed between mesomorphy and 3 RM bench press (r = 0.560, p < 0.001), mesomorphy and 3 RM back squat (r = 0.550, p = 0.001) and between mesomorphy and minimum power output (r = 0.357, p = 0.033). Negative correlations were observed between ectomorphy and 3 RM bench press (r = -0.381, p = 0.022), and ectomorphy and 3 RM back squat (r = -0.336, p = 0.045). Individual regression analysis indicated that mesomorphy was the best predictor of 3 RM bench press performance, with 31.4% of variance in 3 RM bench press performance accounted for by the mesomorphy rating (p < 0.001). A combination of mesomorphy and ectomorphy best predicted 3 RM back squat performance (R^2^ = 0.388, p < 0.04). Around one third of strength performance is predicted by somatotype-assessed physique in physically active males. This could have important implications for the identification of those predisposed to perform well in sports containing strength-based movements and prescription of training programmes.

## Introduction

A somatotype rating gives a categorisation of physique by using measures relating to body shape and composition, assessing adiposity (fatness), musculo-skeletal robustness, and linearity or slenderness. Somatotype “expresses genetic determinism, observed from the morpho-constitutional point of view” [1] and can be identified by assigning a three-numeral rating representing endomorphy, mesomorphy and ectomorphy [2]. In short, the somatotype gives a holistic quantification of the morphology and characteristics of the human body [3]. Authors have recognised the potential application of somatotype analysis to identify talented performers and in the design of training programmes [4].

Anthropometric dimensions influence the ability to perform physical activity [5]. In the athletic population, specific physiques, particularly somatotypes based on the dominant number on the three-numeral rating, have been associated with success in sporting competitions [6–9]. Whilst research has demonstrated that exercise and diet can influence an individual’s somatotype [10], heritability levels of somatotype appear to be moderate [11] to high [12]. Thus, whilst somatotype may be altered, there may be a limit to the magnitude of change.

It is generally not understood whether training for sports brings about physical changes [9], or whether individuals with existing morphological traits become most successful if they enter specific sports [13]. It may even be a combination of both factors, and may be a result of bi-directional relationships between genetics and the environment as suggested by Gottlieb’s [14] theory of probabilistic epigenesis. Given the strength of heritability of somatotype components suggested by Peeters *et al*. [12] and the suggestion by many that somatotype and performance are related, it is necessary to establish the relation between somatotype and aspects of performance in a more comprehensive manner. The suggestion that somatotype itself accounts for up to 65% of the variance in physical fitness tests in adult sportsmen [15] further strengthens the somatotype-performance observation.

Successful athletes in many sports appear to have high mesomorphy ratings, demonstrating strong musculo-skeletal development [7]. In general, larger muscles are able to produce higher strength outputs [15], which can lead to superior anaerobic performance. Many studies have established the link between absolute and task specific strength or power and mesomorphy [1, 4, 8, 15, 16]. However, none of these studies investigate how the magnitude of the other ratings influence performance alongside mesomorphy. Ectomorphy and endomorphy have often been found to explain some of the variance in performance where body propulsion is important such as in explosive leg power [4, 16,17], the association being a positive one with ectomorphy and a negative one with endomorphy. However, low scores in ectomorphy can be advantageous in strength movements where short levers are preferable [6]. Previous research has also demonstrated differences in training-related hormone concentrations both at rest and post-exercise between somatotypes that may help to explain differences in anaerobic performance [18].

The combination of individual somatotype components has received some interest recently. Changes in one somatotype element have been demonstrated to result in changes in another in adolescents over time [19]. Willgoose & Rogers [20] observed a similar pattern in 153 University students. They indicated that mesomorphs with higher endomorphic components were likely to have lower strength and physical fitness index scores than those with lower endomorphic components. Song, Claessens, Lefevre and Beunen [21] and Peeters *et al*. [12] observed that the three somatotype components share genes and environmental factors that contribute to more than 70% of the total variance of each component. They therefore concluded that somatotype should be subject to multivariate analysis rather than looking at each component separately.

The aim of this study, therefore, was to assess the relations between components of somatotype and anaerobic performance using both singular and multivariate analyses. It was hypothesised that there would be a significant relation between multiple components of somatotype and anaerobic performance, and that somatotype as a three-numeral rating could be used as a predictor for anaerobic performance factors.

## Methods

### Subjects

Thirty-six physically active males (mean ± standard deviation age 26.0 ± 9.8 years; body mass 79.5 ± 12.9 kg; height 1.82 ± 0.07 m) were recruited to the study via convenience sampling. Adverts were posted to institutional platforms such as emails and noticeboards at local university facilities, sports clubs and leisure centres. This research was approved by the Institutional Ethics Committee of University of Winchester on 21st February 2013. All subjects were provided with an information sheet and consent form, detailing the purpose of the study and their right to withdraw at any time without any disadvantage of any kind, prior to the start of testing. As such subjects provided written informed consent to participate in the study.

### Methodology

Subjects’ anthropometric profiles were measured by a Level 3 International Society for the Advancement of Kinanthropometry (ISAK) anthropometrist using ISAK protocols [22]. Mean technical error of measurement for skinfolds was 2.12% and for all other measures 0.16%. Intra-class correlation coefficient (ICC) was 1.00 for all measures. Standing height (Seca 213 stadiometer, Birmingham, UK: Seca), body mass (Seca Quadra 808 digital scales, Birmingham, UK: Seca), biceps, triceps, subscapular, iliac crest, supraspinale, abdominal, front thigh and medial calf skinfolds (Harpenden skinfold calipers, Southam, UK: HAB International), upper arm girth (flexed and tensed) and medial calf girth (Cescorf anthropometry tape, Porto Alegre, Brazil: Cescorf), and bi-epicondylar humerus and bi-epicondylar femur breadth (Holtain bone calipers, Pembrokeshire, UK: Holtain) were measured for each participant. Data from the anthropometric assessments were used to calculate somatotype values using the Heath-Carter anthropometric somatotype equations [23]. Mean (± standard deviation) somatotype for the group was: endomorphy 3.4 (± 1.8), mesomorphy 4.5 (± 1.5), ectomorphy 2.6 (± 1.6).

Each participant completed a strength assessment to determine their 3 repetition maximum (3 RM) for bench press and back squat. Due to the mixed experience of participants in weight-lifting it was decided that a 3 RM would be the safest method of testing near-maximal strength, whilst also being a reliable method compared to 1RM in both trained and untrained participants [24]. The 3 RM testing followed guidelines provided by ACSM [25] for 1 RM testing but terminated when the participant could only complete 3 repetitions. Subjects initially completed a 5-minute steady paced cycle and a series of submaximal repetitions of both bench press and back squat in order to warm-up. An initial load was placed on the bar based upon the participant’s perceived ability from previous experience and the participant was required to complete as many repetitions as possible with this load. Following a rest period of 3–5 minutes, the load was increased by 2.5–20 kg (dependent on how many repetitions had been achieved in the previous attempt) and the exercise repeated. When the participant could only complete 3 repetitions of that exercise, the load on the bar was recorded as the 3 RM. Where possible, final 3 RM for each exercise was determined within 4 trials. This was achieved for 80% of participants for both exercises, and 100% of participants for at least one of the exercises.

On a separate day, with at least 48 hours rest subjects completed a submaximal incremental protocol on a cycle ergometer (SRM Training System, Julich, Germany: SRM), starting at 70 W and increasing by 30 W every 5 minutes until blood lactate concentration (extracted from the fingertip) was in excess of 4 mmol.L^-1^ for two stages (Biosen C-Line, Cardiff, UK: EKF Diagnostics). Following a 15 minute rest period subjects then completed to a further incremental protocol to exhaustion. The protocol commenced at the power output 60 W below their final power output on the previous test and increased by 5 W every 15 s. Average power output over the final 60 s of the protocol was calculated as each individual participant’s maximal aerobic power (MAP).

On a further separate occasion with at least another 48 hours rest, subjects completed a maximal sprint cycle. Subjects completed a 10-minute warm-up prior to the test (5 minutes at 100 W and 5 minutes at 60% of individual MAP measured during the previous session) and had a capillary blood sample collected from their fingertip for lactate concentration analysis, pre-test, immediately and 5-minutes post-test. The maximal sprint cycle test involved subjects completing a maximum effort for 30 s on a cycle ergometer (Monark 894E Peak, Sverige, Sweden: Monark) against a resistance of 7.5% body mass [26]. Peak, mean, and minimum power output and time to peak power output were obtained from the computer software linked to the cycle ergometer (Monark ATS Software, Sverige, Sweden: Monark). Fatigue index was calculated as a percentage using the drop in power post peak divided by the peak power and multiplied by one hundred as follows:

$$\mathrm{Fatigue}\;\mathrm{Index}\;(\%)=(\frac{Peak\;power\;(W)-Minimum\;Power\;(W)}{Peak\;Power})\;x\;100$$ (1)

A summary of the participant‘s demographics for anthropometry and performance is shown in Table 1.

**Table 1:** Mean and standard deviation anthropometric and performance data for study participants (N = 36).

Measure	Mean	Standard Deviation
**Height (m)**	1.82	0.07
**Body Mass (kg)**	79.5	12.9
**Endomorphy**	3.4	1.8
**Mesomorphy**	4.5	1.5
**Ectomorphy**	2.6	1.6
**3 RM Bench Press (kg)**	61.0	18.1
**3 RM Back Squat (kg)**	89.6	27.5
**Peak Power Output (W)**	1014.6	196.8
**Mean Power Output (W)**	690.3	105.9
**Minimum Power Output (W)**	424.2	109.6
**Time to peak power (s)**	2.4	1.4
**Fatigue Index (%)**	57.3	11.8

### Statistical analysis

A Shapiro-Wilk test was used to determine that all data were normally distributed. A Pearson correlation analysis was completed to compare somatotype ratings for endomorphy, mesomorphy and ectomorphy with 3 RM bench press, 3 RM back squat, peak power output, mean power output, minimum power output, time to peak power and fatigue index and assessed using Cohen’s [27] correlation thresholds of 0.1 (small), 0.3 (medium) and 0.5 (large). Where significant correlations were observed (p < 0.05), individual regression analysis was completed for each measured variable using the relevant somatotype categories as predictors. All statistical analysis was carried out using IBM SPSS for Windows (IBM Corp. Released 2013. IBM SPSS Statistics for Windows, Version 22.0. Armonk, NY: IBM Corp.).

## Results

**Fig 1:**
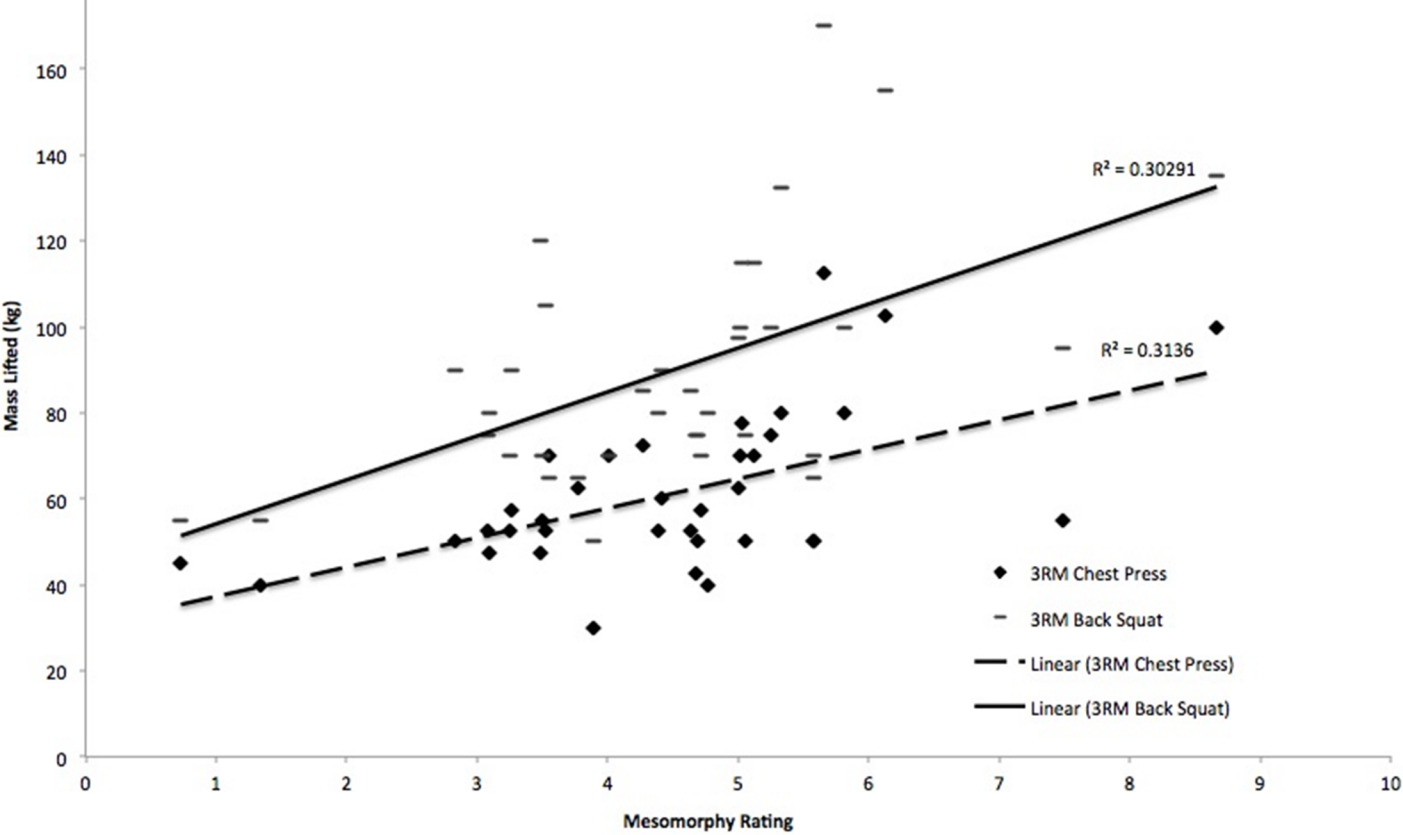
Relation between mesomorphy and 3 RM bench press (p < 0.001) and mesomorphy and 3 RM back squat (p = 0.001).

**Fig 2:**
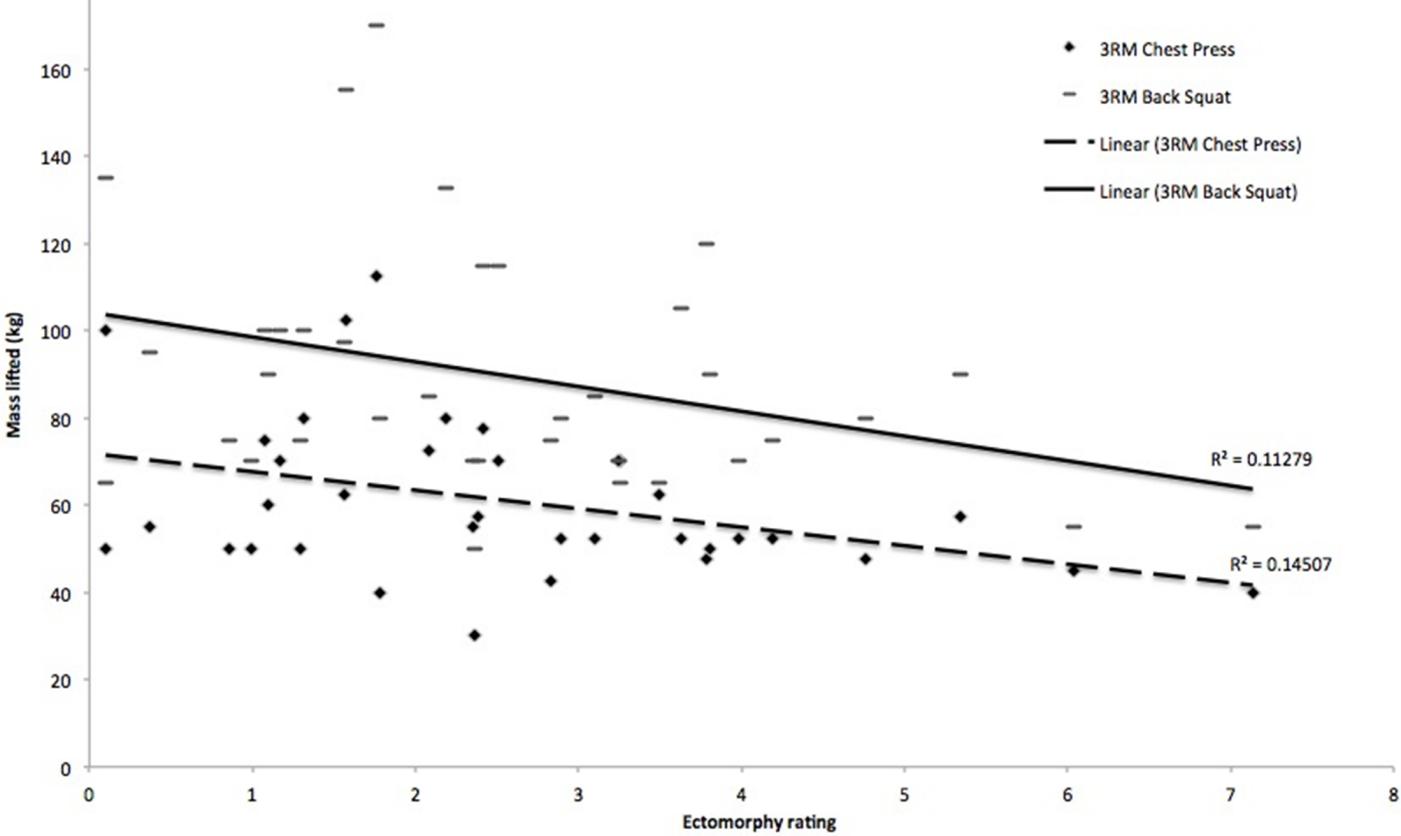
Relation between ectomorphy and 3 RM bench press (p = 0.022) and ectomorphy and 3 RM back squat (p = 0.045).

Significant positive correlations were observed between mesomorphy and 3 RM bench press (r = 0.56, p < 0.000) (Fig 1), mesomorphy and 3 RM back squat (r = 0.55, p = 0.001) (Fig 1), and mesomorphy and minimum power output (r = 0.36, p = 0.033). Significant negative correlations were observed between ectomorphy and 3 RM bench press (r = -0.38, p = 0.022) (Fig 2), and ectomorphy and 3 RM back squat (r = -0.34, p = 0.045) (Fig 2). There were no other significant correlations (p > 0.05) between somatotype ratings and measured variables.

Individual regression analyses indicated that mesomorphy was the best predictor of 3 RM bench press performance (p < 0.001). A combination of mesomorphy and ectomorphy was the best predictor of 3 RM back squat performance. Mesomorphy alone accounted for 30.3% of the variance in 3 RM back squat performance (Step 1; p < 0.05), rising to 38.8% with the addition of the ectomorphy rating into the model (Step 2; p < 0.04). The results from the regression analyses are shown in Table 2. The regression models are as follows:

3RMChestPress(kg)=30.42+(6.85×mesomorphy) (2)

3RMbacksquat(kg)=−24.53+(19.80xmesmorphy)+(10.00xectomorphy) (3)

**Table 2:** Regression model for 3 RM bench press (a), and 3 RM back squat (b) (N = 36).

	B	SE B	β
**(a)**			
**Constant**	30.42	8.15	
**Mesomorphy**	6.85	1.74	0.56*
**(b)**			
**Step 1**			
**Mesomorphy**	10.23	2.66	0.55*
**Step 2**			
**Mesomorphy**	19.80	5.15	1.07*
**Ectomorphy**	10.00	4.68	0.59**

R^2^ = 0.31 for (a). R^2^ = 0.30 for (b) step 1, ΔR^2^ = 0.09 for (b) step 2 (p<0.05).

*p ≤ 0.001.

** p ≤ 0.05.

## Discussion

The aim of this study was to assess the relation between somatotype and anaerobic performance. Mesomorphy demonstrated a large positive significant relation with absolute strength performance in 3 RM bench press and back squat according to Cohen’s [27] definitions on correlation thresholds where 0.5 is considered large. The ectomorphy to strength relation was significant and medium. Endomorphy was not significantly correlated with strength performance. The current study recorded a broad range of somatotype component ratings (endomorphy 1.2–8.3; mesomorphy 0.7–8.7; ectomorphy 0.1–7.1) giving a clear indication of the relation between somatotype and anaerobic performance across the range of different somatotypes. This makes it the first comprehensive study to determine how combined somatotype components predict key aspects of physical performance.

Saha [16] showed that somatotype and body composition variables are important factors in determining leg explosive power. Recognising that power is derived from strength and speed [28] the results of this study appear to confirm those of Saha [16]. Saha [16] found that mesomorphy and ectomorphy components of somatotype were positively correlated with leg explosive power. The mesomorphy relation was slightly smaller than in the current study (r = 0.55) with r = 0.52 for athletes and r = 0.43 for non-athletes. This indicates that the relation between explosive leg power and somatotype is remarkably similar to that between strength and somatotype. This relationship could have important implications for using somatotype and its components to predict performance in power-based sports.

The current study demonstrated a negative correlation between ectomorphy and upper and lower body strength performance. These are similar results to Lewandowska et al. [8] who demonstrated negative correlations between ectomorphy and various combinations of muscle torque measurements in judoists. In contrast to the current study finding no relation between endomorphy and any of the measured components, Saha [16] reported a significant negative correlation between the endomorphy component and leg explosive power, regardless of training experience. The differences between Saha’s [16] study and the current study indicate that ectomorphy and endomorphy could be important in predicting movements where translocation of mass is required, such as in explosive leg power movements [17]. This is supported by results from Busko et al. [4] who observed a significant positive correlation between ectomorphy and maximal power during countermovement jumps, but also between mesomorphy and maximal power during countermovement jumps. The current study minimised the translocation of mass by using single-plane joint movements where endomorphy had no influence and where ectomorphy hindered performance. Low scores in ectomorphy can be advantageous in strength movements where short levers are preferential [6].

Multivariate analyses indicated that mesomorphy was the best component of somtatotype to predict upper body strength, whilst both mesomorphy and ectomorphy predicted lower body strength. In similar findings, Busko et al. [4] indicated that the muscle torques of the upper extremities correlated significantly with the mesomorphy component only. However, in the current study the strongest prediction model of lower body strength combined both mesomorphy and ectomorphy components. In the multivariate analysis, the addition of mesomorphy appears to override the negative relation of ectomorphy to strength, such that being more slender and more muscular combine to create better lower body strength performance. Indeed, the regression model suggests that as mesomorphy increases by 1 unit, 3 RM squat performance will increase by 19.8 kg, and as ectomorphy increases by 1 unit, 3 RM squat performance will increase by 10.0 kg. The combination of high mesomorph and ectomorph somatotype influencing lower body strength may influence decisions in sports where lower body strength is important, with recruitment not just identifying those with a predisposition to muscle mass but also with a strong linearity potentially changing the optimum physique seen in many power based sports.

The current study demonstrated a significant relation between minimum power output and mesomorphy. This indicates that a higher mesomorphy value will result in a higher minimum power value regardless of maximal power output and may be important for events that require maintenance of power output such as speed endurance running and cycling events (e.g. 200 m sprint in athletics or Keirin in track cycling). The current study found no significant relation between any other anaerobic components of sprint cycle performance and individual somatotype ratings. Busko et al. [4] found that power output at varying external loads on a cycle ergometer correlated significantly with all components of somatotype. However, Busko et al.’s [4] study only involved female volleyball athletes, all of whom were centred around the endomorphy and ectomorphy somatotypes, there being very few mesomorphic subjects. This would have resulted in a skew of the data such that correlations would not have represented the full range of possible somatotype values, particularly those high in mesomorphy. The current study indicates that the addition of higher mesomorphic values reduces the relation between somatotype and power output during sprint cycling performance such that physique is not a predicting variable for performance.

While the current study included participants representing a broad range of somatotype ratings, the actual number of participants may have caused some instability in the regression model. Green [29] suggests that the overall fit of a regression model is best tested when the sample size is 50 + 8*k*, where k is the number of predictors; so in the example of this study a regression model using all 3 somatotype ratings would need a sample size of 74 participants. However, Field [30] indicates that this is an oversimplification of the situation and that the sample size needs to be based on the effect size. If Cohen’s [27] benchmark of 0.8 is used for a large effect size and compared to graphs produced by Miles and Shevlin [31] then an ideal required sample size of 40 participants is suggested for 3 predictor variables, very close to the current study sample size.

Establishing the relation between strength and physique could provide important information in the design of training programmes. It is important to recognise that muscular strength performance is also determined by other biological and behavioural variables [17]. In particular, influencing factors upon the remaining two thirds of strength performance in the current study may have included the individual impact of the chosen warm-up [32–34], where some participants chose to stretch and others did not. Further, pre-performance mental state and nutritional status were not assessed in the current study and have previously been demonstrated to influence strength performance [35–39] Indeed, the morphological state of somatotype itself can be considerably influenced by prior exposure to neural, behavioural and environmental events [14]. However, the current study indicates that over a 3rd of both upper and lower body strength performance may be predicted by one or more somatotype components. If Ignjatovic et al.’s [40] argument, that those who are stronger have an advantage in strength training, holds true, then it would seem that those with certain physiques may also have an advantage in strength training since the prediction model suggests that a higher mesomorphy rating relates to higher strength. Any advantage in strength training apportioned to higher mesomorphy ratings could also be related to relations between training-associated hormones (cortisol, ACTH) and somatotype, both at rest and post exercise [18]. Authors have suggested that there is a relation between somatotype and trainability in children [41] and young people [40]. Whilst training will, inevitably, alter some anthropometric characteristics relevant to somatotype, such as body weight and muscle mass, there are others that are determined by genetics (e.g. height and bone breadth) [42]. Due to the high genetic determinability of somatotype (up to 85% [43]), this may mean that strength training responses are specific to physique [42].

## Conclusion

This study has demonstrated a link between somatotype and an aspect of anaerobic performance; strength, with at least one third of strength performance predicted by one or more aspect of somatotype. In particular, it would seem that those who have high mesomorphy values are predisposed to better strength performance. In the lower body, this may also be combined with a higher ectomorphy value. Overall, these findings may have important implications for both the identification of those predisposed to perform well in sports containing strength-based movements and prescription of training programmes in physically active males.

## Supporting information

S1 Raw Data File (XLSX)
